# Histological grade 2 and non-contrast-enhancing phenotype provide prognostic information complementary to DNA methylation classification in *TERT*p-mutant molecular glioblastomas

**DOI:** 10.1186/s40478-026-02269-z

**Published:** 2026-03-04

**Authors:** Hiroyuki Sueyoshi, Kenji Fujimoto, Katsumi Fujita, Kazuhito Tanaka, Hirotaka Inoue, Rin Yamada, Takahiro Yamamoto, Yutaka Nakachi, Jun-ichiro Kuroda, Naoki Shinojima, Kazuya Iwamoto, Yoshiki Mikami, Akitake Mukasa

**Affiliations:** 1 Department of Neurosurgery, Faculty of Life Sciences, Kumamoto University, Kumamoto, Japan; 2 School of Medicine, Kumamoto University, Kumamoto, Japan; 3 Department of Diagnostic Pathology, Kumamoto University Hospital, Kumamoto, Japan; 4 Department of Cell Pathology, Faculty of Life Sciences, Kumamoto University, Kumamoto, Japan; 5 Department of Molecular Brain Science, Graduate School of Medical Sciences, Kumamoto University, Kumamoto, Japan

**Keywords:** Glioblastoma, Molecular GBM, *TERT* promoter mutation, DNA methylation profiling, *WIF-1*

## Abstract

Glioblastoma (GBM), *IDH*-wildtype, is the most common and aggressive primary brain tumor in adults. According to the 2021 WHO classification, *IDH*-wildtype diffuse gliomas with a *TERT* promoter (*TERT*p) mutation, *EGFR* amplification, or combined whole-chromosome 7 gain and 10 loss (+ 7/− 10) are designated as GBM, *IDH*-wildtype, regardless of histological grade, and termed molecular GBM (mol-GBM). Although this redefinition has been widely adopted, the prognostic implications of mol-GBM remain uncertain. To clarify its clinical relevance, we retrospectively analyzed adult patients treated at our institution between 2010 and 2024. We identified 22 cases of *TERT*p-mutant mol-GBM (grade 2, *n* = 7; grade 3, *n* = 15) and compared them with 218 cases of *TERT*p-mutant histologically confirmed GBM (hist-GBM). Overall survival (OS) was assessed using Kaplan–Meier analysis, log-rank test, and propensity score matching (PSM) adjusted for age, preoperative Karnofsky Performance Status, extent of resection, adjuvant therapy, and *MGMT* methylation status. Mol-GBM showed significantly longer OS than hist-GBM after PSM (*p* = 0.019), with grade 2 mol-GBM showing particularly favorable survival (*p* = 0.008). Cox regression analysis revealed mol-GBM as an independent predictor of good prognosis (hazard ratio: 0.37, 95% confidence interval: 0.22–0.62, *p* < 0.001). Across all *TERT*p-mutant GBMs, a non-contrast-enhancing (non-CE) MRI phenotype correlated with significantly longer OS than CE tumors, persisting after PSM (*p* = 0.004). Within mol-GBM, an isolated *TERT*p mutation did not stratify OS, whereas *CDKN2A/B* homozygous deletion predicted worse outcomes (*p* = 0.026). DNA methylation profiling classified most mol-GBM as GBM, *IDH*-wildtype methylation class, and all cases clustered with GBM on t-SNE, confirming molecular proximity to GBM. Integrated methylation and RNA-seq analysis revealed *WIF-1* promoter hypermethylation with reduced expression in grade 3 cases. These findings suggest that histological grade 2 and the non-CE phenotype may provide exploratory prognostic information in *TERT*p-mutant mol-GBM, complementary to DNA methylation classification.

**Supplementary Information:**

The online version contains supplementary material available at 10.1186/s40478-026-02269-z.

## Introduction

Glioblastoma (GBM), the most frequent and lethal primary malignant brain tumor in adults, was substantially redefined in the fifth edition of the World Health Organization (WHO) Classification of Tumors of the Central Nervous System, 5th Edition (WHO CNS5) [28]. In the WHO CNS5, *IDH*-wildtype diffuse astrocytic gliomas that show microvascular proliferation or necrosis, or that harbor a *TERT* promoter (*TERT*p) mutation, *EGFR* amplification, or combined whole-chromosome 7 gain and 10 loss (+ 7/− 10), are designated as GBM, *IDH*-wildtype, WHO grade 4. Among these, tumors that lack microvascular proliferation or necrosis but fulfill one of the molecular criteria (*TERT*p mutation, *EGFR* amplification, or + 7/− 10) are termed “molecular GBM (mol-GBM)”, reflecting GBM with lower-grade histological appearance but with GBM-defining molecular alterations. This is in contrast with the “histological GBM” (hist-GBM), which is defined by classical high-grade morphology [9].

Although this reclassification has been widely adopted, significant debate persists regarding whether mol-GBM truly reflects hist-GBM in terms of clinical presentation, molecular heterogeneity, and prognosis. Regarding survival, published findings have been contradictory. Some cohorts have reported broadly similar outcomes between mol-GBM and conventional hist-GBM [17, 19], even in cases with an isolated *TERT*p mutation [40, 43]. By contrast, other studies, most notably the Paris cohort, found that *IDH*-wildtype grade 2 gliomas with an isolated *TERT*p mutation demonstrated a markedly longer median overall survival (OS) (approximately 88 months) [7]. More recently, a multicenter case series highlighted that in *IDH*-wildtype diffuse gliomas with minimal histological atypia, tumors harboring an isolated *TERT*p mutation do not consistently follow the aggressive clinical trajectory of GBM, emphasizing the need for caution when interpreting limited molecular alterations [34].

Given these uncertainties, there is a critical need to refine the biological and prognostic delineation of mol-GBM, particularly in the context of *TERT*p-mutant tumors. DNA methylation profiling has emerged as a powerful adjunct in glioma classification, often surpassing histology in diagnostic accuracy and prognostic prediction [2, 10, 41]. However, the epigenetic landscape of mol-GBM and its potential to distinguish biologically indolent from aggressive subsets remain incompletely defined.

In this study, we systematically investigated a cohort of *IDH*-wildtype mol-GBM, with particular attention to cases harboring *TERT*p mutations. We compared their clinicopathological and survival characteristics with those of hist-GBM, assessed prognostic determinants within mol-GBM, and explored their DNA methylation profiles. By integrating clinical, molecular, and epigenomic data, we aimed to characterize the heterogeneity of mol-GBM within the current WHO framework and to examine whether histological grade and radiological features provide complementary prognostic information regarding tumors classified as GBM, *IDH*-wildtype.

## Materials and methods

### Patients

We retrospectively identified adult patients (≥ 18 years) who underwent their first surgical treatment for diffuse glioma at Kumamoto University Hospital between January 2010 and March 2024. From this cohort, we selected those with *IDH*1/2-wildtype, *TERT*p mutation, *H3F3A*-wildtype tumors that were histologically classified as WHO grade 2 or 3 (mol-GBM). For OS analysis, we used a comparison cohort of 218 patients diagnosed with histological GBM, *IDH*-wildtype, WHO grade 4 (hist-GBM), and *TERT*p mutant, who were also surgically treated at our institution during the same period. We collected clinical and treatment data, including age at diagnosis, sex, preoperative Karnofsky Performance Status (KPS), extent of resection, radiation dose, initial chemotherapy regimen, and *MGMT* promoter methylation status. Patient survival data were updated to the most recent follow-up when available. Cases were excluded if survival time could not be determined due to loss to follow-up or if molecular analysis could not be completed because of insufficient DNA quantity.

### Definition of non-contrast-enhancing (non-CE) tumors

Non-CE tumors were defined as lesions that showed no gadolinium enhancement or exhibited an enhancing area of < 1 cm^3^ on MRI at initial diagnosis. This definition was based on the report by Karschnia et al., which demonstrated no significant difference in survival outcomes between patients with non-CE GBMs and those with minimally CE GBMs (0–1 cm^3^) (*p* = 0.422; OS: 29 ± 3.1 months vs. 26 ± 5.3 months) [24].

### Histopathological diagnosis

Histopathological diagnoses of mol-GBM and hist-GBM were established by two board-certified neuropathologists at our institution and classified according to the WHO CNS5 criteria. Based on hematoxylin and eosin (H&E) staining, tumors with minimal nuclear atypia, rare or absent mitotic figures, and lacking both necrosis and microvascular proliferation were classified as WHO grade 2. Biopsy specimens with a single mitotic figure warranted classification as grade 3. Tumors exhibiting nuclear atypia and mitotic activity but without necrosis or microvascular proliferation were classified as WHO grade 3, whereas those showing either necrosis or microvascular proliferation were classified as WHO grade 4 [3, 8, 11, 12, 15]. For mol-GBM cases, histological grading was reassessed in accordance with the WHO CNS5 criteria, including retrospectively collected cases.

### Extraction of genomic DNA and total RNA

For molecular analyses, genomic DNA and total RNA were extracted from tumor tissues obtained during surgery and stored at –80 ℃. DNA was isolated using the QIAamp DNA Mini Kit (QIAGEN), and RNA was isolated using the RNeasy Mini Kit (QIAGEN), according to the manufacturer’s instructions (RRID: SCR_008539).

### *IDH*, *TERT*p, *BRAF,* and *FGFR1* mutation analysis

The presence of *IDH*1 (R132) and *IDH*2 (R172), *TERT*p (C228T and C250T), *BRAF* V600E, and *FGFR1* K656E mutations was analyzed using Sanger sequencing or pyrosequencing of genomic DNA extracted from tumor tissue, as previously reported [5]. Primer sequences are provided in Supplementary Table S1.

### Copy number analysis

Copy number alterations (CNAs), including *EGFR* amplification, + 7/–10 signature, *CDKN2A/B* homozygous deletion (HD), and *PDGFRA* amplification, were analyzed using DNA methylation profiling with the Infinium MethylationEPIC v2.0 BeadChip (Illumina, San Diego, CA, USA) and Multiplex Ligation-dependent Probe Amplification (MLPA) with SALSA MLPA kits (probe mixes P105 and P088, MRC-Holland), as previously reported [23]. DNA methylation profiling was performed in mol-GBM cases with sufficient tumor material, whereas MLPA was used for copy number assessment in mol-GBM cases with limited tumor material and in all hist-GBM cases. Chromosome 7 gain and chromosome 10 loss were inferred by evaluating *EGFR* gain and *PTEN* loss, respectively. MLPA data were analyzed for copy number variation using Coffalyser.Net software (MRC-Holland).

### *MGMT* methylation analysis

The methylation status of the *MGMT* promoter was assessed through pyrosequencing using genomic DNA extracted from tumor tissue. A cutoff value of ≥ 10% methylation was used to define *MGMT* promoter methylation, as previously reported [26, 33]. Results obtained from pyrosequencing were fully concordant with those obtained from DNA methylation profiling.

### DNA methylation profiling and t-distributed stochastic neighbor embedding (t-SNE) analysis

DNA methylation profiling was conducted using the Infinium MethylationEPIC v2.0 BeadChip Kit. Data processing was performed using Minfi (RRID: SCR_012830) [6], and differential methylation was conducted using limma (RRID: SCR_010943) [1, 21]. Classification of central nervous system tumors was performed using the Molecular Neuropathology classifier (https://www.molecularneuropathology.org/mnp/). For dimensionality reduction, our samples were jointly analyzed with reference data from GSE109381 (RRID: SCR_005012) [10] using t-SNE implemented with the Rtsne package [31]. Detailed preprocessing steps are described in the Supplementary Methods.

### RNA sequencing (RNA-seq)

RNA-seq was performed on high-quality RNA (RIN ≥ 7) using poly(A)-selected, strand-specific libraries, sequenced on the NovaSeq X Plus platform. Lowly expressed genes were filtered using a CPM threshold [32], and differential expression analysis was performed with DESeq2 (RRID: SCR_015687) [30]. Detailed preprocessing steps are described in the Supplementary Methods.

### Integrative analysis of DNA methylation profiling and RNA-seq

Integrated analysis was conducted by focusing on promoter-associated CpG probes [37] for methylation and CPM thresholds [32] for RNA-seq data. Differentially methylated and expressed genes were combined for downstream analyses. Detailed preprocessing steps are described in the Supplementary Methods.

### Quantitative polymerase chain reaction (qPCR)

qPCR was performed to assess gene expression. Detailed protocols, including reagents, instrumentation, and primer sequences, are provided in the Supplementary Methods and Supplementary Table S1.

### Statistical analysis

Differences in age distribution were examined using Student’s t-test. Associations between categorical variables were evaluated using Fisher’s exact test. OS was defined as the time from the date of the initial imaging study suggesting the presence of a tumor to the date of death from any cause or the date of the last follow-up. Patients who were alive at the time of the last follow-up were censored at that date. OS was estimated using the Kaplan–Meier method, and survival curves were compared using the log-rank test. In addition, OS was compared using propensity score matching (PSM) based on age, KPS, extent of resection, adjuvant therapy, and *MGMT* methylation status. To avoid multicollinearity, correlations among candidate variables were assessed using the phi coefficient for categorical variables and Pearson’s correlation coefficient for continuous variables. Variables with strong intercorrelation (|r| or φ ≥ 0.7) were not simultaneously included in the multivariate Cox regression model. A *p*-value < 0.05 was considered statistically significant. All statistical analyses were performed using R version 4.4.2 (RRID: SCR_001905) and GraphPad Prism 9 (GraphPad Software Inc., USA, RRID: SCR_002798).

## Results

### Patient characteristics

Between January 2010 and March 2024, a total of 579 adult patients (≥ 18 years) underwent initial surgical treatment for WHO grade ≥ 2 diffuse gliomas at our institution. Among them, 442 cases were identified as *IDH*1/2-wildtype. Six cases were excluded from analysis: three due to insufficient tumor tissue for DNA extraction, which prevented confirmation of *IDH* or *TERT*p mutation status, and three due to lack of follow-up data. Of the remaining cases, 240 *IDH*-wildtype gliomas harbored *TERT*p mutations. Within this subset, 22 cases were histologically diagnosed as WHO grade 2 or 3 and were classified as mol-GBM, while the remaining 218 cases were classified as hist-GBM. The clinical characteristics of the mol-GBM and hist-GBM cohorts are summarized in Table 1. Detailed case-by-case clinical, radiological, histopathological, molecular, and survival data for the mol-GBM and hist-GBM cases are provided in Supplementary Tables S2 and S3, respectively. The male-to-female ratio was significantly higher in the mol-GBM group, with 19 of 22 patients being male (*p* = 0.005). There was no significant difference in adjuvant therapy between the two groups (*p* = 0.174). However, biopsy-only procedures were significantly more frequent in the mol-GBM group than in the hist-GBM group (*p* < 0.001). At the initial presentation, 18 of the 22 mol-GBM cases showed no gadolinium enhancement on contrast-enhanced MRI. The frequency of non-contrast enhancement was significantly higher in mol-GBM than in hist-GBM (*p* < 0.001).

**Table 1:** Patient characteristics

	mol-GBM (*n* = 22)	grade2 (*n* = 7)	grade3 (*n* = 15)	hist-GBM (*n* = 218)	*p* value
*Age at diagnosis*					
Median (range)	67(38–81)	72(55–81)	66(38–81)	66(23–96)	0.735
*Sex*					
Male	19	7	12	114	0.005
Female	3	0	3	104	
*KPS*					
≧80	14	5	9	114	0.373
< 80	8	2	6	104	
*Surgery*					
Gross total removal	4	3	1	100	< 0.001
Partial resection	4	1	3	87	
Biopsy	14	3	11	31	
*Adjuvant therapy*					
CRT	20	6	14	202	0.174
RT only	1	1	0	2	
Chemotherapy only	0	0	0	10	
No	1	0	1	3	
NA	0	0	0	1	
*Initial MRI enhancement status*					
CE	4	0	4	207	< 0.001
non-CE	18	7	11	7	

*p*-values were calculated using Student’s t-test or Fisher’s exact test between mol-GBM and hist-GBM. CE, contrast enhanced; CRT, chemoradiotherapy; hist-GBM, histologically confirmed glioblastoma; KPS, Karnofsky Performance Status; mol-GBM, molecular glioblastoma; NA, not available; RT, radiotherapy

### Molecular profiles of mol-GBM

**Fig. 1:**
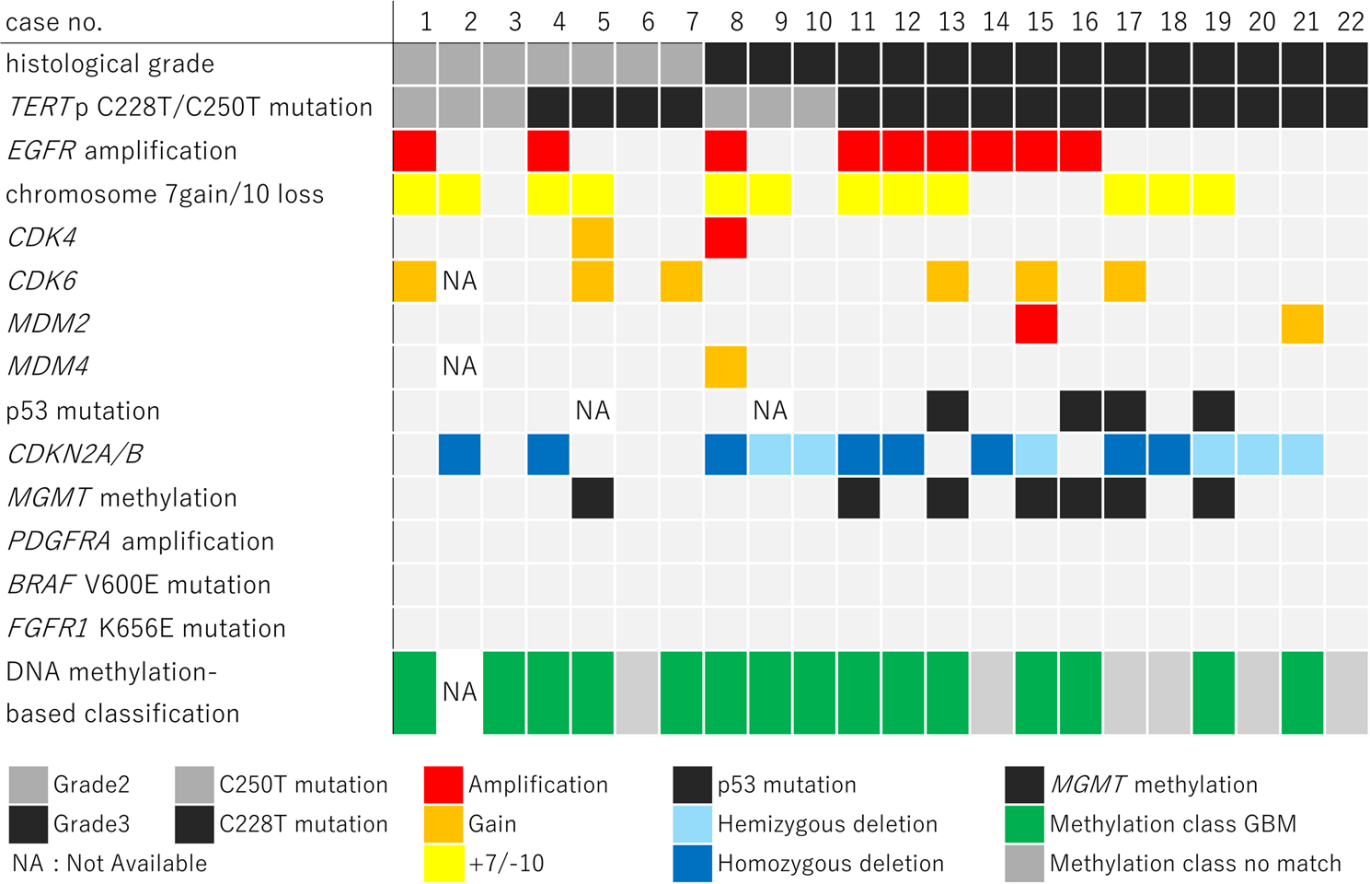
Oncoprint summarizing genetic alterations, copy number alterations, histological grade, and DNA methylation-based classifications in individual mol-GBM cases. mol-GBM, molecular glioblastoma

Figure 1 summarizes the molecular profiles of mol-GBM. Among the 22 cases of mol-GBM, seven were histologically classified as grade 2 and 15 as grade 3. CNAs were evaluated by DNA methylation profiling in 21 cases and by MLPA in one case. *EGFR* amplification was observed in two grade 2 cases (28.6%) and seven grade 3 cases (46.7%). The + 7/–10 signature was present in four grade 2 cases (57.1%) and eight grade 3 cases (53.3%). *CDKN2A/B* HD was identified in two grade 2 cases (28.6%) and six grade 3 cases (40.0%). *MGMT* promoter methylation was detected in one grade 2 case (14.3%) and six grade 3 cases (40.0%). None of these differences between grade 2 and 3 tumors was statistically significant. An isolated *TERT*p mutation was observed in three grade 2 cases (42.9%) and four grade 3 cases (26.7%).

### Non-CE tumors and prognosis in *TERT*p mutant GBM

Among the 22 mol-GBM cases, 18 (82%) were non-CE on the initial MRI, including seven grade 2 and 11 grade 3 tumors. Of these, six of seven grade 2 and 10 of 11 grade 3 tumors eventually developed CE during follow-up; one grade 3 case was lost to follow-up. In contrast, of the 218 hist-GBM cases, 207 were CE, and seven were non-CE at diagnosis, while four patients could not undergo contrast-enhanced MRI due to contraindications such as hemodialysis or pacemaker implantation (Table 1). Among the initially non-CE cases, we examined the latency to the first appearance of contrast enhancement to evaluate whether non-CE mol-GBM exhibits a prolonged pre-enhancement phase compared with hist-GBM. To minimize potential confounding by treatment-related imaging changes, this analysis was restricted to patients who underwent surgical resection during the non-CE phase and did not receive radiotherapy or chemotherapy before contrast enhancement was observed. In this selected cohort, the median interval from the initial MRI to the first radiographic appearance of contrast enhancement was 251 days in mol-GBM (*n* = 4) compared with 59 days in hist-GBM (*n* = 6) (Supplementary Fig. S1). Although the number of evaluable cases was limited, individual patient-level data consistently demonstrated a longer CE-free interval in mol-GBM, with all evaluable mol-GBM cases showing a longer latency than the median latency observed in hist-GBM.

**Fig. 2:**
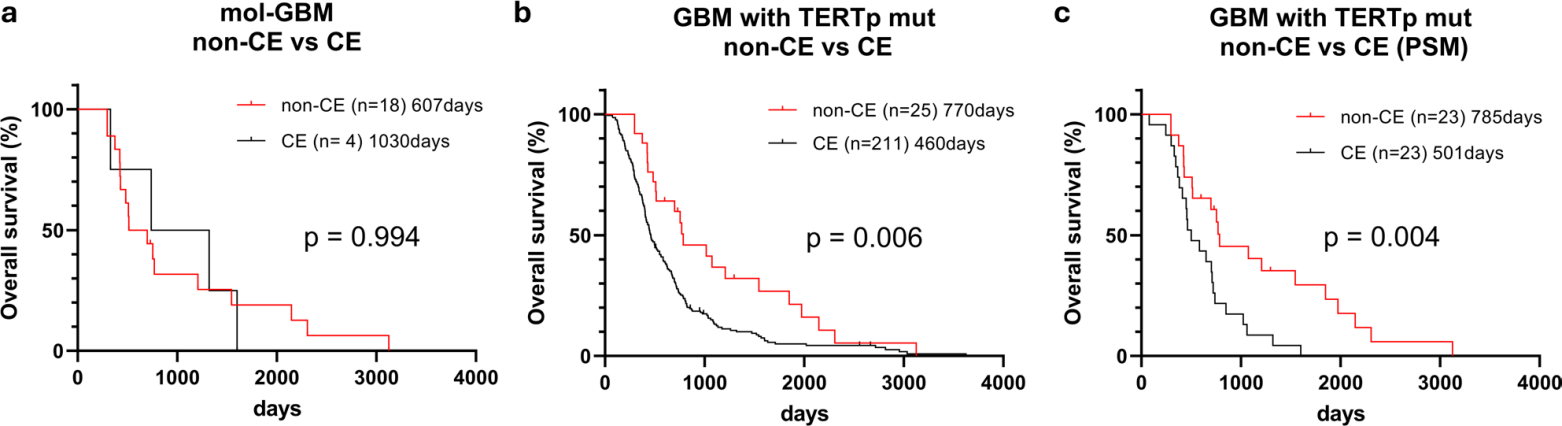
Kaplan–Meier survival curves comparing OS between CE and non-CE GBM. Median OS in days is indicated in each panel. Subgroup analyses: **a** non-CE versus CE mol-GBM on initial MRI; **b** non-CE versus CE GBM (mol-GBM and hist-GBM) with *TERT*p mutation; **c** non-CE versus CE GBM (mol-GBM and hist-GBM) with *TERT*p mutation after PSM. CE, contrast-enhancing; hist-GBM, histologically confirmed glioblastoma; mol-GBM, molecular glioblastoma; OS, overall survival; PSM, propensity score matching; *TERT*p: *TERT* promoter

We compared OS between the 18 non-CE and four CE mol-GBM cases at initial MRI and found no significant difference (*p* = 0.994; median OS: 607 vs. 1,030 days; Fig. 2a). However, when analyzing all *TERT*p-mutant GBMs (mol-GBM and hist-GBM combined), the 25 non-CE cases had significantly longer OS than the 211 CE cases (*p* = 0.006; median OS: 770 vs. 460 days; Fig. 2b). This survival advantage for non-CE tumors remained significant even after PSM for age, KPS, extent of resection, adjuvant therapy, and *MGMT* methylation status (*p* = 0.004; median OS: 785 vs. 501 days; Fig. 2c).

### Mol-GBM is associated with longer OS compared with hist-GBM

**Fig. 3:**
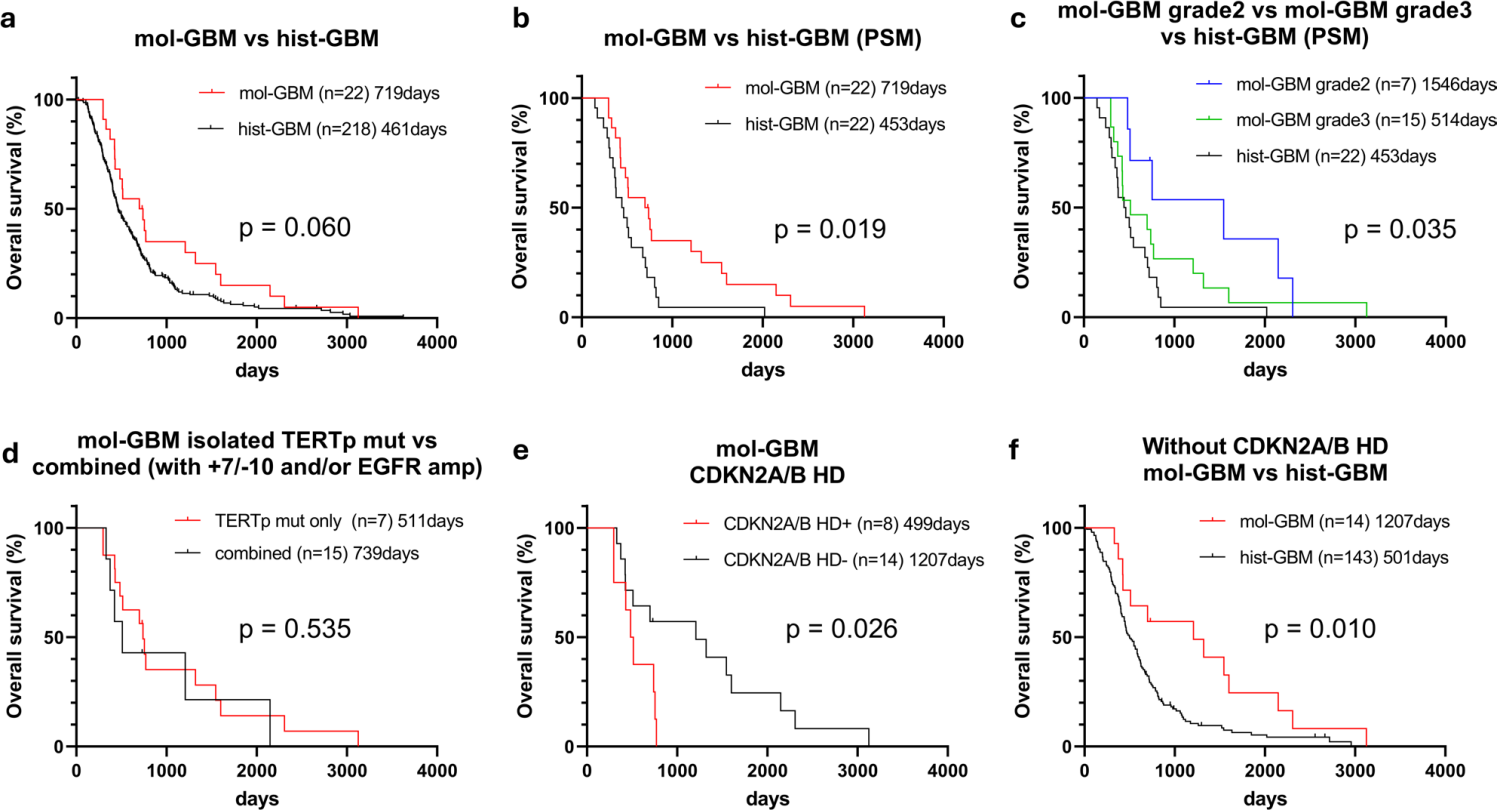
Kaplan–Meier survival curves comparing OS between mol-GBM and hist-GBM. Median OS in days is indicated in each panel. Subgroup analyses: **a** mol-GBM versus hist-GBM; **b** mol-GBM versus hist-GBM after PSM; **c** mol-GBM grade 2 versus mol-GBM grade 3 vs. hist-GBM after PSM; **d** mol-GBM with isolated *TERT*p mutation (no *EGFR* amp or 7+/10–) versus cases with additional molecular features; **e** mol-GBM with vs. without *CDKN2A/B* HD. **f** mol-GBM vs. hist-GBM after exclusion of cases with *CDKN2A/B* HD. hist-GBM, histologically confirmed glioblastoma; HD, homozygous deletion; mol-GBM, molecular glioblastoma; OS, overall survival; PSM, propensity score matching; *TERT*p, *TERT* promoter

OS was compared between mol-GBM with *TERT*p mutation (*n* = 22) and hist-GBM with *TERT*p mutation (*n* = 218). OS tended to be longer in the mol-GBM group, although the difference was not statistically significant (*p* = 0.060; median OS: 719 vs. 461 days; Fig. 3a). After PSM based on age, KPS, extent of resection, adjuvant therapy, and *MGMT* methylation status, OS was significantly longer in the mol-GBM group than in the hist-GBM group (*p* = 0.019; median OS: 719 vs. 453 days; Fig. 3b). To further examine the prognostic impact of mol-GBM, we performed multivariate Cox regression analysis including possible confounding variables such as age, preoperative KPS, extent of resection, adjuvant therapy, and *MGMT* promoter methylation status. Due to the strong association between mol-GBM and non-CE at initial MRI (φ = 0.74, *p* < 0.001), these two variables were not entered simultaneously into the multivariate Cox regression model to avoid multicollinearity. The results showed that mol-GBM was an independent predictor of good prognosis (hazard ratio [HR]: 0.37, 95% confidence interval [CI]: 0.22–0.62, *p* < 0.001) in addition to gross total resection (GTR), adjuvant chemoradiotherapy, and *MGMT* promoter methylation (Table 2).

**Table 2:** Multivariate Cox regression analysis comparing molecular and histological GBM

Variable	HR(95% Cl)	*p* value
*Tumor type*		
mol-GBM	0.37 (0.22–0.62)	< 0.001
hist-GBM	Ref	–
Age (per 1-year increase)	1.01 (0.995–1.02)	0.205
Preoperative KPS (per 10-point increase)	0.996 (0.99–1.004)	0.325
*Extent of resection*		
Non-GTR	1.93 (1.44–2.59)	< 0.001
GTR	Ref	–
*Adjuvant therapy*		
Non-CRT	2.20 (1.24–3.91)	0.012
CRT	Ref	–
*MGMT promoter methylation*		
Unmethylated	2.26 (1.69–3.01)	< 0.001
Methylated	ref	–

CE, contrast enhanced; CI, confidence interval; CRT, chemoradiotherapy; GTR, gross total removal

hist-GBM, histologically confirmed glioblastoma; HR, hazard ratio; KPS, Karnofsky performance status; mol-GBM, molecular glioblastoma

We further subdivided the mol-GBM cohort by histological grade and compared OS among mol-GBM grade 2, mol-GBM grade 3, and hist-GBM after PSM. Kaplan–Meier analysis demonstrated a significant difference in OS among mol-GBM grade 2, mol-GBM grade 3, and hist-GBM (global log-rank, *p* = 0.035; Fig. 3c). In exploratory pairwise comparisons, mol-GBM grade 2 showed significantly longer survival than hist-GBM (*p* = 0.008, Bonferroni-adjusted *p* = 0.024; median OS: 1546 vs. 453 days). Within the mol-GBM group, there was no significant difference in OS between cases with isolated *TERT*p mutation (that is, without *EGFR* amplification or chromosome 7 + /10– alterations) and those with additional *EGFR* amplification or + 7/–10 alterations (combined group) (*p* = 0.535; median OS: 511 vs. 739 days; Fig. 3d). When further subdivided by histological grade, comparisons between isolated *TERT*p mutation and the combined group showed no significant difference (Supplementary Fig. S2a, b). Similarly, OS did not significantly differ between mol-GBM cases with and without + 7/–10 alterations (Supplementary Fig. S2c) or between those with and without *EGFR* amplification (Supplementary Fig. S2d).

In contrast to these findings, OS was significantly shorter in mol-GBM cases with *CDKN2A/B* HD than in those without HD (*p* = 0.026; median OS: 499 vs. 1,207 days; Fig. 3e). In contrast, *CDKN2A/B* HD was not associated with OS within the hist-GBM cohort (Supplementary Fig. S2e). To evaluate the potential confounding effect of *CDKN2A/B* HD on survival, we performed subgroup analyses stratified by *CDKN2A/B* status. When the analysis was restricted to cases without *CDKN2A/B* HD, OS differed significantly between mol-GBM and hist-GBM (Fig. 3f). Patients with mol-GBM without *CDKN2A/B* HD (*n* = 14) showed significantly longer OS than those with hist-GBM without *CDKN2A/B* HD (*n* = 143), with median OS of 1207 days versus 501 days, respectively (*p* = 0.010). Among cases with *CDKN2A/B* HD, no significant difference in OS was observed between mol-GBM (*n* = 8) and hist-GBM (*n* = 75) (*p* = 0.810; median OS: 499 vs. 416 days; Supplementary Fig. S2f).

We next examined whether the survival advantage associated with the non-CE phenotype could be explained by an imbalance in *CDKN2A/B* status. Among *TERT*p-mutant GBMs without *CDKN2A/B* HD, non-CE tumors (*n* = 17) were associated with significantly longer OS than CE tumors (*n* = 137) (*p* = 0.001; median OS: 1074 vs. 480 days; Supplementary Fig. S2g).

Finally, OS did not differ significantly between mol-GBM cases with and without *MGMT* promoter methylation (*p* = 0.169; median OS: 1,320 vs. 511 days; Supplementary Fig. S2h).

### DNA methylation profiling clusters mol-GBM with GBM

**Fig. 4:**
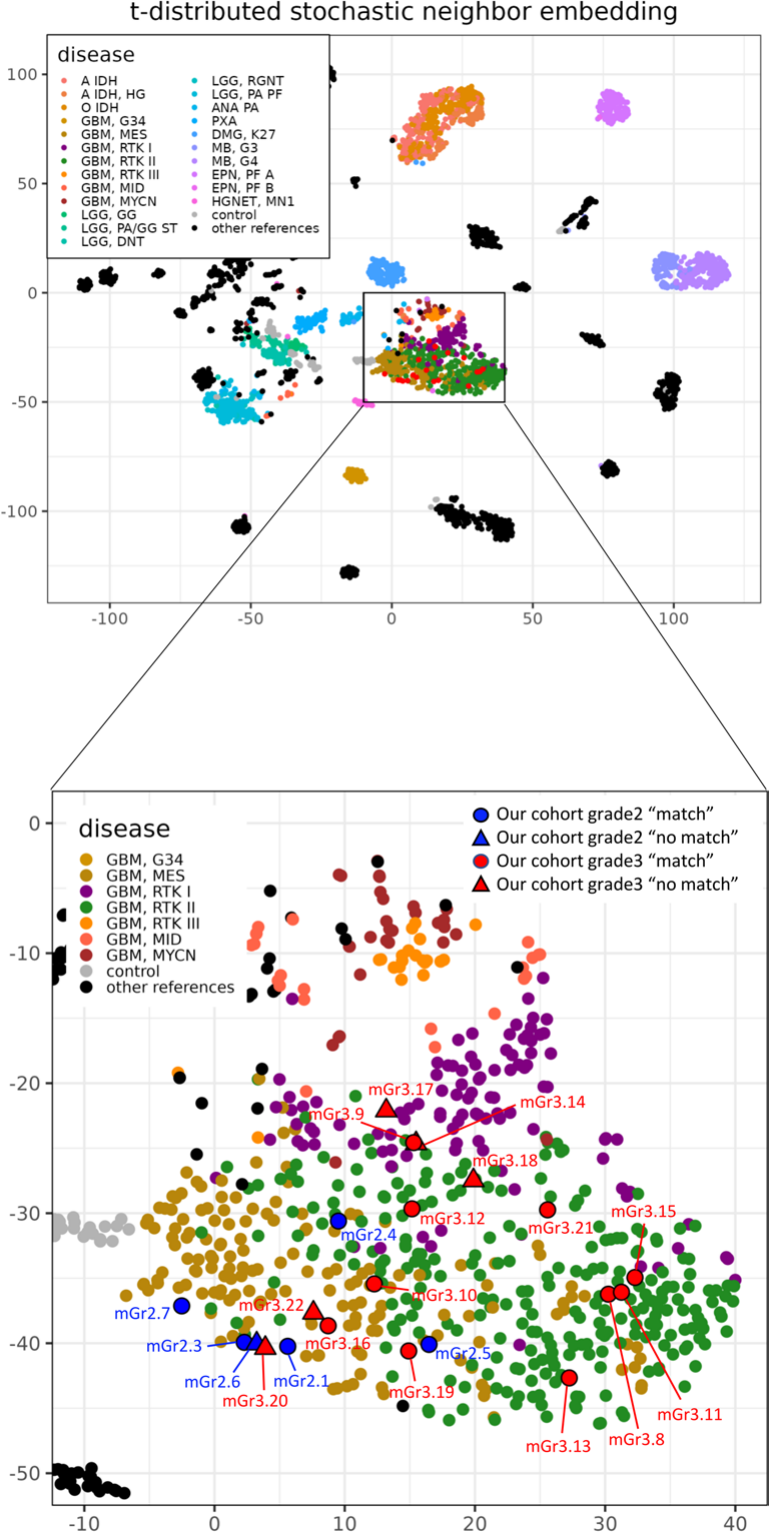
t-SNE plot generated by integrating our samples with the reference dataset from NCBI GSE109381 (3905 samples). Each dot represents a single sample. Our cohort is shown in blue (mol-GBM grade 2) and red (mol-GBM grade 3), labeled as mGr2.x and mGr3.x, respectively (for example, mGr2.1 indicates mol-GBM grade 2, sample no. 1). ● indicates samples that were classified as “match” by DNA methylation profiling, whereas ▲ indicates samples classified as “no match.” mol-GBM: molecular glioblastoma, t-SNE: t-distributed stochastic neighbor embedding, NCBI: National Center for Biotechnology Information

Genome-wide DNA methylation analysis was performed in 21 cases (grade 2: *n* = 6; grade 3: *n* = 15). The results obtained with the Molecular Neuropathology classifier (v12.8) are summarized in Table 3. Among the six grade 2 cases, five matched the GBM, *IDH*-wildtype class, including four assigned to the mesenchymal (MES) subtype. One case had a calibrated score of 0.78 for the MES subtype but did not reach the matching threshold, and one case was classified as “no match.” Among the 15 grade 3 cases, 10 matched the GBM, *IDH*-wildtype class. Within this group, three matched the MES subtype, one matched RTK 1, and five matched RTK 2. One additional case had a calibrated score of 0.82 for the MES subtype but did not reach the threshold. The remaining five cases were categorized as “no match.” To further explore clustering, t-SNE analysis was performed using 3,905 reference samples from NCBI GSE109381. All 21 mol-GBM cases, including the six “no match” cases, clustered within the GBM group (Fig. 4).

**Table 3:** DNA methylation–based classification of mol-GBM cases

Case	ID	Methylation classes (Highest level > = 0.3, lower levels > = 0.1, all of lowest level)	Calibrated score (subclass)	Interpretation (subclass)
1	mGr2.1	Mc Glioblastoma, IDH Wildtype, Mesenchymal Subtype	0.99	match
2	mGr2.2	NA	NA	NA
3	mGr2.3	Mc Glioblastoma, IDH Wildtype, Mesenchymal Subtype	0.92	match
4	mGr2.4	Mc Glioblastoma, IDH Wildtype, Mesenchymal Subtype	0.98	match
5	mGr2.5	Glioblastoma, IDH Wildtype (Glioblastoma, IDH Wildtype, Mesenchymal Type)	0.99 (0.78)	match (no match)
6	mGr2.6	Glioblastoma, IDH Wildtype	0.66	no match
7	mGr2.7	Mc Glioblastoma, IDH Wildtype, Mesenchymal Subtype	0.95	match
8	mGr3.8	Mc Glioblastoma, IDH Wildtype, Rtk2 Subtype	0.99	match
9	mGr3.9	Mc Glioblastoma, IDH Wildtype, Rtk1 Subtype	0.93	match
10	mGr3.10	Mc Glioblastoma, IDH Wildtype, Mesenchymal Subtype	0.99	match
11	mGr3.11	Mc Glioblastoma, IDH Wildtype, Rtk2 Subtype	0.99	match
12	mGr3.12	Mc Glioblastoma, IDH Wildtype, Rtk2 Subtype	0.99	match
13	mGr3.13	Mc Glioblastoma, IDH Wildtype, Rtk2 Subtype	0.99	match
14	mGr3.14	Glioblastoma, IDH Wildtype, Rtk1 Type	0.4	no match
15	mGr3.15	Mc Glioblastoma, IDH Wildtype, Rtk2 Subtype	0.98	match
16	mGr3.16	Mc Glioblastoma, IDH Wildtype, Mesenchymal Subtype	0.97	match
17	mGr3.17	Diffuse High Grade Neuroepithelial Tumour [adult Type]	0.52	no match
18	mGr3.18	Glioblastoma, IDH Wildtype	0.36	no match
19	mGr3.19	Glioblastoma, IDH Wildtype (Glioblastoma, IDH Wildtype, Mesenchymal Type)	0.90 (0.82)	match (no match)
20	mGr3.20	Ependymal Tumours	0.3	no match
21	mGr3.21	Mc Glioblastoma, IDH Wildtype, Mesenchymal Subtype	0.99	match
22	mGr3.22	Diffuse High Grade Neuroepithelial Tumour [adult Type]	0.44	no match

mGr2.x, molecular GBM grade 2; mGr3.x, molecular GBM grade 3

### Integrative analysis of DNA methylation profiling and RNA-seq

**Fig. 5:**
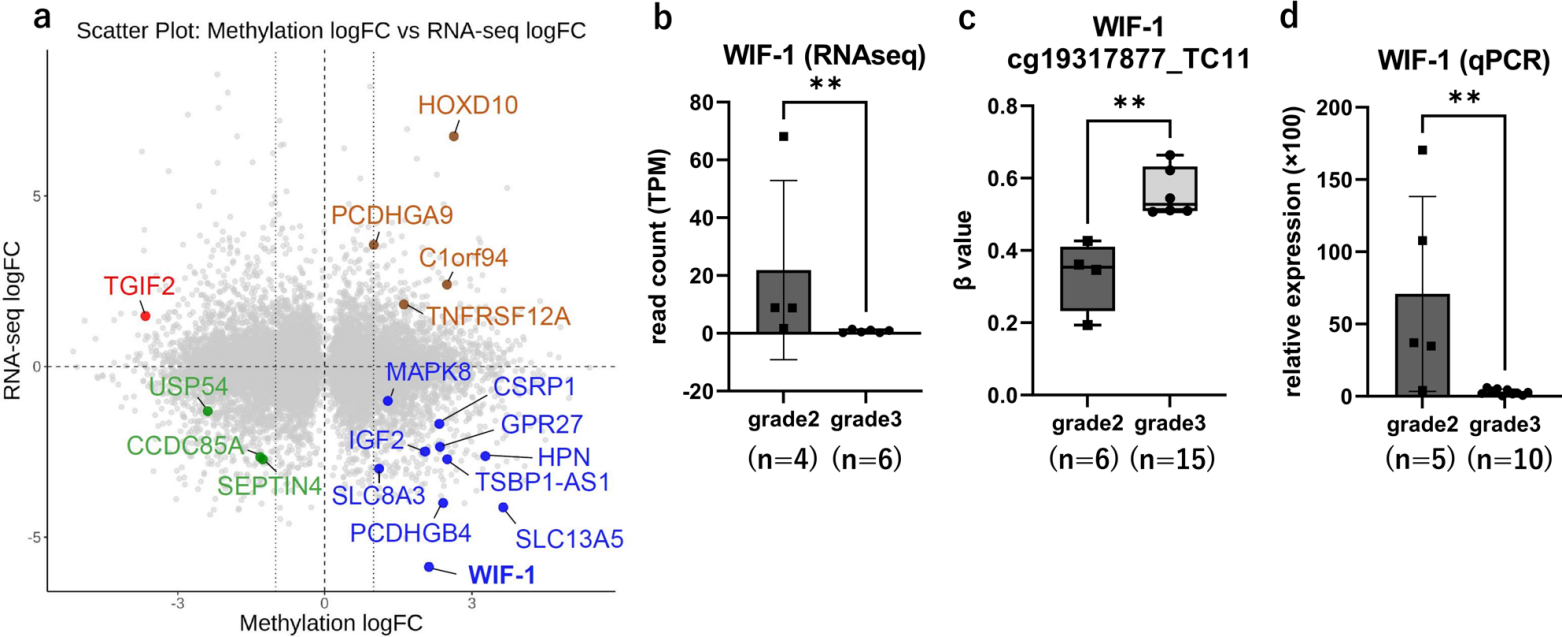
Integrated analysis of DNA methylation and RNA expression in grade 2 vs. grade 3 mol-GBM. **a** Scatter plot of methylation logFC versus RNA logFC. The x-axis represents methylation logFC, where negative values indicate lower methylation (M-values) in grade 3 mol-GBM, and positive values indicate higher methylation in grade 3 than in grade 2 mol-GBM. The y-axis represents RNA logFC, with positive values indicating genes upregulated in grade 3 mol-GBM and negative values indicating genes upregulated in grade 2 mol-GBM. Genes meeting significant thresholds (methylation adjusted *p* < 0.05, |methylation logFC|> 1; RNA-seq adjusted *p* < 0.05, |RNA-seq logFC|> 1) are color-coded according to their regulatory pattern: Red: hypomethylated and upregulated in grade 3 mol-GBM. Blue: hypermethylated and downregulated in grade 3 mol-GBM. Green: hypomethylated and downregulated in grade 3 mol-GBM. Brown: hypermethylated and upregulated in grade 3 mol-GBM. Grey: no significant difference between grade 2 and grade 3 mol-GBM. **b** RNA-seq analysis of *WIF-1* expression (TPM) showing significantly lower levels in grade 3 mol-GBM than in grade 2 mol-GBM (Wilcoxon rank-sum test, *p* = 0.0095). Each dot represents an individual sample; bars indicate mean ± standard deviation. **c** Comparison of *WIF-1* promoter methylation β-values between grade 2 and grade 3 mol-GBM, probe cg19317877_TC11. Bars indicate mean ± standard deviation (Wilcoxon rank-sum test, *p* = 0.008). **d** qPCR analysis confirming downregulation of *WIF-1* expression in grade 3 mol-GBM compared with that in grade 2 mol-GBM (relative expression; Wilcoxon rank-sum test, *p* = 0.005). Each dot represents an individual sample; bars indicate mean ± standard deviation. qPCR, quantitative polymerase chain reaction; mol-GBM, molecular glioblastoma

RNA-seq was successfully performed in 10 cases (mol-GBM grade 2: *n* = 4; grade 3: *n* = 6), which were subjected to integrated analysis with DNA methylation profiles. A two-group comparison was conducted between mol-GBM grade 2 and grade 3. Genes were extracted for analysis if they met all of the following criteria and visualized in a scatter plot (Fig. 5a):

1. a significant difference in methylation score (M-value) with adjusted *p* < 0.05 and an absolute log fold change (|Methylation logFC|) > 1, and
2. a significant difference in RNA expression (CPM-filtered) with adjusted *p* < 0.05 and |RNA logFC|> 1.

From this integrated analysis, we identified *WIF-1*, a gene that was hypermethylated and downregulated in grade 3 compared with grade 2 mol-GBM. RNA-seq confirmed that *WIF-1* expression was significantly lower in grade 3 (*n* = 6) than in grade 2 (*n* = 4) cases (*p* = 0.0095, Fig. 5b). Consistently, analysis of a representative *WIF-1* promoter probe (cg19317877_TC11) demonstrated significantly higher β-values in grade 3 (*n* = 15) than in grade 2 (*n* = 6) cases (*p* = 0.008, Fig. 5c). To validate these findings, *WIF-1* expression was further assessed by qPCR in grade 2 (*n* = 5) and grade 3 (*n* = 10) cases, again confirming significantly lower expression in grade 3 (*p* = 0.005, Fig. 5d). RNA-seq and qPCR analyses showed a significant positive correlation in *WIF-1* expression levels (Spearman’s rank correlation, ρ = 0.714, *p* = 0.046).

## Discussion

This study aimed to explore clinically relevant heterogeneity within WHO-defined *TERT*p–mutant mol-GBM, particularly with respect to histological grade and radiological presentation. We examined the clinical, molecular, and radiological characteristics of *TERT*p–mutant mol-GBM within the current WHO framework. Compared with hist-GBM harboring the same mutation, mol-GBM cases showed longer OS after adjusting for major clinical confounders. However, this observation should be interpreted cautiously due to the limited sample size.

Within the mol-GBM cohort, histological grade appeared to be associated with the outcome, with grade 2 cases showing a trend toward longer survival than grade 3 cases, whereas an isolated *TERT*p mutation did not confer a prognostic advantage. In contrast, *CDKN2A/B* HD was associated with shorter survival, and exclusion of *CDKN2A/B* HD revealed significantly longer OS in mol-GBM cases than in hist-GBM cases, highlighting the influence of *CDKN2A/B* status on prognostic discrimination between these groups.

Radiologically, the absence of CE at presentation was uncommon but clinically informative. Across the combined cohort of *TERT*p–mutant GBM (mol-GBM and hist-GBM) cases, non-CE tumors were associated with longer survival than CE tumors, even after accounting for major clinical and molecular confounders.

Genome-wide DNA methylation profiling demonstrated that all analyzed mol-GBM cases, including those classified as “no match,” clustered within the GBM, *IDH*-wildtype methylation group, supporting their alignment with high-grade glioma biology despite histological heterogeneity.

The concept of mol-GBM was first introduced in cIMPACT-NOW update 3 and was formally adopted in the 2021 WHO classification of CNS tumors [9, 28]. Although mol-GBM is currently classified as GBM regardless of histological grade, its prognostic implications remain uncertain. Several groups have reported outcomes comparable to hist-GBM [17, 19, 40, 43], whereas others have suggested more favorable survival in subsets stratified by histology or molecular profile [7, 34]. Berzero et al. analyzed 47 patients with *IDH*-wildtype diffuse astrocytoma, grade 2, and demonstrated that histological grade retained prognostic significance in mol-GBM. Specifically, patients with grade 2 tumors (*n* = 47, median OS, 59 months) showed significantly longer survival than those with grade 3 tumors (*n* = 255; median OS, 19 months, *p* < 0.0001). Moreover, patients with grade 2 tumors harboring isolated *TERT*p mutations (*n* = 16) had particularly favorable outcomes (median OS, 88 months) [7]. In contrast, Wijnenga et al. examined 132 patients with mol-GBM identified across three Dutch centers and found no survival differences either between histological grades (grade 2: *n* = 60; grade 3: *n* = 15; grade 4: *n* = 6) or between mol-GBM and 197 cases of hist-GBM. Within grade 2 tumors, survival did not differ between those with isolated *TERT*p mutation and those with additional *EGFR* amplification or + 7/− 10 alterations (*p* = 0.59) [43]. An earlier study of the same group of 74 *IDH*-wildtype diffuse low-grade gliomas highlighted the molecular heterogeneity of these tumors: 39 cases with + 7/− 10 alterations (nearly all with *TERT*p mutation) had a dismal prognosis, whereas 14 cases with isolated *TERT*p mutation exhibited significantly shorter survival than the + 7/− 10 group (*p* = 0.024), suggesting that isolated *TERT*p mutation is not uniformly favorable [42]. More recently, Priesterbach et al. reported 56 patients with mol-GBM presenting minimal histological change. Among them, 12 patients with isolated *TERT*p mutation demonstrated significantly longer survival than 40 patients harboring additional *EGFR* amplification or + 7/− 10 alterations (*p* = 0.007) [34].

In our cohort of 22 patients with *TERT*p-mutant mol-GBM (grade 2: n = 7; grade 3: *n* = 15), PSM analysis indicated that grade 2 cases tended to show longer OS than hist-GBM cases, supporting the potential prognostic significance of histological grade. In contrast to Priesterbach et al., we observed no significant survival difference between patients with isolated *TERT*p mutations and those with combined alterations (*EGFR* amplification and/or + 7/− 10), irrespective of histological grade. These findings suggest that an isolated *TERT*p mutation is not necessarily associated with improved outcome and that, at least in our series, pathological grade appeared to be more closely associated with survival than molecular features. Larger, multi-institutional studies with harmonized histopathological review and molecular assessment will be required to resolve these discrepancies and to clarify the clinical significance of isolated *TERT*p mutations in this challenging subgroup of gliomas.

In our series of 240 *IDH*-wildtype, *TERT*p-mutant GBM (including both mol-GBM and hist-GBM), non-CE presentation on baseline MRI was uncommon but clinically informative. Of the 236 patients with preoperative contrast-enhanced MRI, 25 (11%) were non-CE or minimally enhancing; of these, 18 (72%) fulfilled the mol-GBM criteria (7 grade 2 and 11 grade 3). Non-CE tumors demonstrated significantly longer OS than CE tumors (*p* = 0.006), and this association persisted after PSM (*p* = 0.004). These findings are concordant with prior reports. Foltyn-Dumitru et al. identified non-CE in 12.5% (44/352) of *IDH*-wildtype GBM, with 55% (24/44) classified as mol-GBM. Non-CE tumors were less likely to exhibit classical histological hallmarks, microvascular proliferation (39% vs. 94%) and necrosis (25% vs. 92%), and were associated with significantly longer median OS (27.2 vs. 14.7 months, *p* = 0.0049) [16]. Similarly, Karschnia et al. reported non-CE in 7.4% (98/1,323) of *IDH*-wildtype GBM, of which 63% met mol-GBM criteria and demonstrated superior survival for non-CE cases than for CE cases (32 ± 3.3 vs. 20 ± 1.9 months, *p* = 0.008) [24].

*CDKN2A/B* HD is a well-established adverse prognostic marker in *IDH*-mutant astrocytomas and, under the WHO CNS5 framework, is sufficient to be assigned grade 4 irrespective of histology [4, 8, 29, 39]. However, its prognostic significance in *IDH*-wildtype gliomas has not been fully clarified. Fujimoto et al. reported a trend toward shorter survival in *IDH*-wildtype, *TERT*p-mutant lower-grade gliomas with *CDKN2A/B* HD (*p* = 0.079), suggesting a potential negative effect in this molecular context [17]. In line with these observations, our analysis demonstrated that among mol-GBM cases harboring *TERT*p mutations, *CDKN2A/B* HD-positive tumors were associated with significantly shorter OS compared with HD-negative tumors. In contrast, *CDKN2A/B* HD did not show a significant association with OS within the hist-GBM cohort. Exclusion of *CDKN2A/B* HD revealed a significant survival advantage of mol-GBM over hist-GBM, highlighting that *CDKN2A/B* status substantially influences the detectability of prognostic differences between these groups. Together, these results indicate that the prognostic impact of *CDKN2A/B* HD in *IDH*-wildtype, *TERT*p-mutant GBM is context-dependent and may influence the interpretation of histological and molecular subclassification.

Capper et al. proposed a classification system for gliomas based on DNA methylation profiles, which has since gained widespread acceptance and growing clinical and research importance [2, 10, 41]. In our cohort of 21 mol-GBM cases, DNA methylation profiling classified 15 tumors as GBM, *IDH*-wildtype methylation class, whereas six were categorized as “no match.” Among the matched samples, seven tumors corresponded to the GBM, *IDH*-wildtype, MES Subtype. Although MES, RTK1, and RTK2 subtypes display distinct pathobiological and clinical characteristics [13, 35], most studies have not demonstrated survival differences across these groups [14, 25]. Importantly, all 21 cases clustered within the GBM group in the t-SNE analysis, indicating their alignment with high-grade glioma biology despite histological heterogeneity. In our cohort, survival appeared to differ between mol-GBM and hist-GBM even though all analyzed mol-GBM cases aligned with the GBM, *IDH*-wildtype methylation group. These findings suggest that clinically relevant heterogeneity may exist within the WHO-defined GBM and that histological grade and radiological phenotype may provide prognostic information complementary to DNA methylation classification. These findings contrast with the report by Priesterbach et al., who examined 12 cases of isolated *TERT*p-mutant mol-GBM with minimal histological atypia [34]. In their series, only one case matched the adult-type GBM, *IDH*-wildtype methylation class, whereas most clustered with rare or lower-grade-like methylation subgroups. A potential explanation for why the isolated *TERT*p mutation was not associated with worse prognosis in our series is that the DNA methylation classification patterns differed from those observed in their cohort. These findings underscore the continued relevance of histopathological assessment, even as DNA methylation-based classification gains wider use. Among the six cases classified as “no match,” all still clustered within the GBM group in the t-SNE analysis, suggesting that their low calibrated scores may have reflected undersampling. Notably, grade 2 and grade 3 cases showed a tendency toward partially overlapping but non-identical spatial distributions in the t-SNE plot. While substantial overlap remained, this visual pattern suggests the possibility of grade-associated differences in DNA methylation profiles and should be interpreted in an exploratory manner. Validation in a larger cohort will be required to confirm these findings.

In our cohort, mol-GBM exhibited a clear male predominance, with 19 of 22 patients (86%) being male. This tendency was particularly apparent among histological grade 2 cases, all of which occurred in men. Although glioblastoma is generally known to occur more frequently in men, a similar sex bias has been reported by Berzero et al., who observed that 24 of 29 patients (83%) with histological grade 2 mol-GBM were male [7]. Although the mechanisms underlying this sex difference remain unclear, the consistent male predominance across independent cohorts implies a possible intrinsic susceptibility that warrants further investigation.

Integrated analysis of DNA methylation profiling and RNA-seq revealed increased promoter methylation with concomitant downregulation of *WIF-1* in grade 3 cases. WIF-1 is a secreted antagonist that binds directly to Wnt proteins and inhibits their activity [22]. Activation of the Wnt signaling pathway has been implicated in promoting GBM cell proliferation and invasion [18, 27, 44]. Yang et al. reported that *WIF-1* expression decreases with increasing histological grade in astrocytomas and that this downregulation is associated with promoter methylation [45]. Although gliomas are highly heterogeneous and their proliferative and invasive capacities cannot be attributed solely to *WIF-1* expression, the epigenetic silencing of this gene may represent one mechanism contributing to the more aggressive biological behavior and poorer prognosis observed in a subset of mol-GBM.

### Limitations

Some limitations should be acknowledged. First, this study was retrospective in design and was conducted at a single institution, which may limit the generalizability of the findings. Multi-institutional studies with larger sample sizes and standardized pathological and molecular evaluations are warranted to validate these results. Second, the prolonged OS observed in a subset of mol-GBM cases raises the possibility of early detection (lead-time) bias. Earlier diagnosis may advance the starting point of survival measurement without necessarily reflecting a true biological survival advantage. However, most mol-GBM cases in our cohort were not incidentally detected but were identified following tumor-related neurological symptoms, such as seizures or focal neurological deficits, which prompted diagnostic imaging and tissue sampling. Although these tumors radiologically resembled lower-grade gliomas, sampling was clinically justified and consistent with standard diagnostic practice. While most non-CE mol-GBM cases eventually developed contrast enhancement, many exhibited a prolonged pre-enhancement phase. Third, 13 of the 22 cases in this study were diagnosed through navigation-guided biopsy. Histopathological interpretation based on biopsy specimens can be influenced by the sampling site, and undersampling of areas containing hist-GBM components may have led to the underestimation of the tumor grade. Such biopsy-related bias may therefore have influenced survival estimates in this cohort. Additionally, biopsies often yield limited tissue samples or samples with low tumor cell content [36], which can result in case exclusion and may ultimately compromise the robustness of the findings. Finally, the extent of resection was not systematically assessed. Because a greater extent of resection has been associated with improved prognosis in GBM [20, 38], GTR might have conferred even longer OS in some cases.

## Conclusion

Our findings suggest that, within the WHO-defined *TERT*p-mutant mol-GBM, histological grade, particularly histological grade 2, may be associated with a trend toward longer OS than hist-GBM cases. However, given the small sample size (*n* = 22, including only seven grade 2 cases), these observations should be cautiously interpreted and considered exploratory. Among the mol-GBM cases, six were not assigned to any defined DNA methylation class (“no match”). While DNA methylation profiling remains an important and valuable tool for molecular classification, our results also highlight the continued relevance of histopathological assessment. An integrated diagnostic approach that combines both molecular and histological information may provide a more accurate basis for the prognostication of the WHO-defined GBM. Furthermore, the absence of contrast enhancement on initial MRI may signal a more favorable prognosis in *IDH*-wildtype GBM. Finally, reduced expression of *WIF-1* in mol-GBM, associated with promoter hypermethylation, may contribute to tumor aggressiveness and warrant further investigation.

## Supplementary Information

Below is the link to the electronic supplementary material.

Supplementary Material 1.

Supplementary Material 2.

Supplementary Material 3.

## Data Availability

The datasets generated and/or analysed during the current study are available from the corresponding author on reasonable request.

## Abbreviations

CE: Contrast-enhancing
CI: Confidence interval
CNA: Copy number alteration
CGGA: Chinese glioma genome atlas
CNS: Central nervous system
CRT: Chemoradiotherapy
GBM: Glioblastoma
GTR: Gross total removal
HD: Homozygous deletion
hist-GBM: Histologically confirmed glioblastoma
HR: Hazard ratio
IDH: Isocitrate dehydrogenase
KPS: Karnofsky performance status
MLPA: Multiplex ligation-dependent probe amplification
mol-GBM: Molecular glioblastoma
OS: Overall survival
PSM: Propensity score matching
qPCR: Quantitative polymerase chain reaction
RNA-seq: RNA sequencing
RT: Radiotherapy
t-SNE: T-distributed stochastic neighbor embedding
TCGA: The cancer genome atlas
*TERT*p: *TERT* promoter
WHO: World Health Organization
WIF-1: Wnt inhibitory factor-1
