# Supplementary material for: Histological grade 2 and non-contrast-enhancing phenotype provide prognostic information complementary to DNA methylation classification in TERTp-mutant molecular glioblastomas
Source: Acta Neuropathol Commun. 2026 Mar 4;14:88. doi: 10.1186/s40478-026-02269-z (PMC13069819; doi:10.1186/s40478-026-02269-z)
Supplement: Supplementary file 1 — Supplementary Material 1. [file 40478_2026_2269_MOESM1_ESM.docx]

**Supplementary Methods**

**DNA Methylation Profiling and t-SNE Analysis**

DNA methylation profiling of tumor samples was performed using the Infinium MethylationEPIC v2.0 BeadChip Kit. Raw output data (IDAT files) were quality-checked using Genome Studio (Illumina, RRID:SCR_010973). Data were normalized, and probes associated with SNPs as well as those derived from the X and Y chromosomes were removed. Batch effects were corrected prior to analysis. Methylation levels at each CpG site were calculated as M-values using the Minfi Bioconductor package (version 1.52.1, RRID:SCR_012830) [2]. Differential methylation analysis was performed with the limma package (version 3.62.2, RRID:SCR_010943), applying linear modeling and variance estimation using the empirical Bayes method [1, 5]. For classification of central nervous system tumors, IDAT files were uploaded to the Molecular Neuropathology classifier (https://www.molecularneuropathology.org/mnp/). As a reference dataset, IDAT files from 3,905 samples registered in the NCBI Gene Expression Omnibus (GEO; accession GSE109381, RRID:SCR_005012) were downloaded and used [3]. Since our samples were analyzed on the Infinium MethylationEPIC v2.0 BeadChip, whereas GSE109381 data were generated on the Infinium HumanMethylation450K BeadChip, common probes between the two platforms were extracted for analysis. Among these, the top 1,000 most variable probes, as determined by standard deviation, were selected [4]. Our samples and the reference samples were then plotted using t-SNE [7], implemented with the Rtsne package (version 0.17) with 2,500 iterations and a perplexity parameter of 35.

**RNA Sequencing**

The quality of extracted RNA was assessed using the Agilent 2100 Bioanalyzer (Agilent Technologies, Santa Clara, CA, USA; RRID: SCR_018043), and samples with an RNA Integrity Number (RIN) ≥ 7 were selected for RNA sequencing (RNA-seq). Library preparation and sequencing were outsourced to Rhelixa (Tokyo, Japan). mRNA was purified using a poly(A) selection method, and strand-specific libraries were constructed using the dUTP method. The libraries were PCR-amplified and sequenced on the Illumina NovaSeq X Plus platform, generating 150 bp paired-end reads. Sequencing data were obtained in FASTQ format and processed for downstream analysis. Lowly expressed genes were excluded using a count-per-million (CPM) filter, retaining those with CPM > 1 in at least two samples [8]. Differentially expressed gene (DEG) analysis was then performed using the DESeq2 package in R (RRID: SCR_015687) [6].

**Integrative Analysis of DNA Methylation Profiling and RNA Sequencing**

To enable integrated analysis, the respective datasets were preprocessed as follows. For DNA methylation profiling data, probes were filtered to include only those located in promoter-associated regions (TSS1500, TSS200, and 5′UTR) [9]. When a single CpG probe was annotated with multiple genes, a serial number was appended to the probe ID to distinguish each gene-specific annotation. For each gene, the probe that showed a statistically significant difference in M-values between mol-GBM grade 2 and grade 3 (adjusted p < 0.05) and had the largest absolute log fold change (logFC) was selected, and its M-value was used to represent the methylation level of that gene. For RNA-seq, DEG data obtained after CPM filtering (CPM > 1 in at least two samples) and subsequent analysis with DESeq2 (RRID: SCR_015687) were used for integration with DNA methylation data.

**Quantitative PCR (qPCR)**

Total RNA was reverse-transcribed into cDNA using the FastGene™ Scriptase II cDNA Synthesis 5× ReadyMix OdT Oligo dT (Genetics, Tokyo, Japan), following the manufacturer’s instructions. qPCR was performed using the THUNDERBIRD SYBR™ qPCR Mix (TOYOBO, Osaka, Japan), and fluorescence signals were detected using the ViiA™ 7 Real-Time PCR System (Thermo Fisher Scientific). *GAPDH* was used as the reference gene. As a normal brain control, total RNA extracted from normal adult human brain tissue (product number: R1234035-50) was purchased from BioChain Institute Inc. (Newark, CA, USA). Gene expression levels were quantified using the relative quantification method. The sequences of each primer are listed in Supplementary Table 1.

References

1 Ajuyah P, Mayoh C, Lau LMS, Barahona P, Wong M, Chambers H et al (2023) Histone H3-wild type diffuse midline gliomas with H3K27me3 loss are a distinct entity with exclusive EGFR or ACVR1 mutation and differential methylation of homeobox genes. Sci Rep 13:3775. <https://doi.org/10.1038/s41598-023-30395-4>

2 Aryee MJ, Jaffe AE, Corrada-Bravo H, Ladd-Acosta C, Feinberg AP, Hansen KD et al (2014) Minfi: a flexible and comprehensive Bioconductor package for the analysis of Infinium DNA methylation microarrays. Bioinformatics 30:1363-1369. <https://doi.org/10.1093/bioinformatics/btu049>

3 Capper D, Jones DTW, Sill M, Hovestadt V, Schrimpf D, Sturm D et al (2018) DNA methylation-based classification of central nervous system tumours. Nature 555:469-474. <https://doi.org/10.1038/nature26000>

4 Fujimoto K, Arita H, Satomi K, Yamasaki K, Matsushita Y, Nakamura T et al (2021) TERT promoter mutation status is necessary and sufficient to diagnose IDH-wildtype diffuse astrocytic glioma with molecular features of glioblastoma. Acta Neuropathol 142:323-338. <https://doi.org/10.1007/s00401-021-02337-9>

5 Hutarew G, Holzl D, Schiefer T, Langwieder CK, Alinger-Scharinger B, Schlicker HU et al (2022) Methylome Profiling of PD-L1-Expressing Glioblastomas Shows Enrichment of Post-Transcriptional and RNA-Associated Gene Regulation. Cancers (Basel) 14. <https://doi.org/10.3390/cancers14215375>

6 Love MI, Huber W, Anders S (2014) Moderated estimation of fold change and dispersion for RNA-seq data with DESeq2. Genome Biol 15:550. <https://doi.org/10.1186/s13059-014-0550-8>

7 Maaten Lvd (2008) Visualizing Data using t-SNE. Journal of Machine Learning Research

8 Moradi Marjaneh M, Kirk EP, Patrick R, Alankarage D, Humphreys DT, Del Monte-Nieto G et al (2023) Quantitative trait and transcriptome analysis of genetic complexity underpinning cardiac interatrial septation in mice using an advanced intercross line. Elife 12. <https://doi.org/10.7554/eLife.83606>

9 Salz L, Seitz A, Schafer D, Franzen J, Holzer T, Garcia-Prieto CA et al (2023) Culture expansion of CAR T cells results in aberrant DNA methylation that is associated with adverse clinical outcome. Leukemia 37:1868-1878. <https://doi.org/10.1038/s41375-023-01966-1>
