# Supplementary material for: Histological grade 2 and non-contrast-enhancing phenotype provide prognostic information complementary to DNA methylation classification in TERTp-mutant molecular glioblastomas
Source: Acta Neuropathol Commun. 2026 Mar 4;14:88. doi: 10.1186/s40478-026-02269-z (PMC13069819; doi:10.1186/s40478-026-02269-z)
Supplement: Supplementary file 2 — Supplementary Material 2. [file 40478_2026_2269_MOESM2_ESM.docx]

**
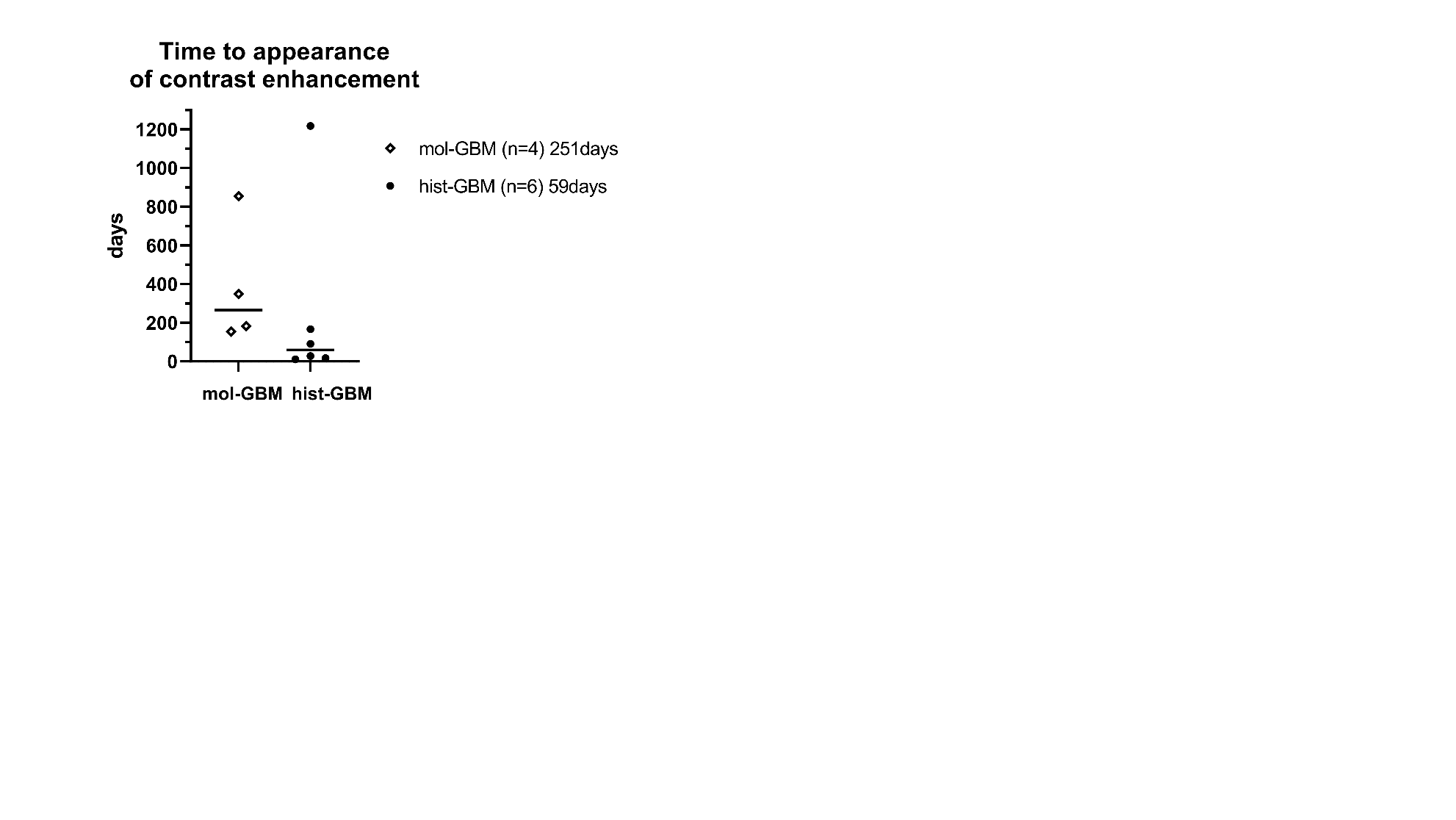
**

**Supplementary Fig. S1** Time to appearance of contrast enhancement in initially non-CE GBM. Scatter plot showing the interval from the initial MRI to the first radiographic appearance of contrast enhancement in mol-GBM and hist-GBM. Horizontal bars indicate median values (251 days for mol-GBM and 59 days for hist-GBM). Only patients who underwent surgical resection during the non-CE phase and did not receive radiotherapy or chemotherapy before CE emergence were included. Individual data points represent single patients. CE: contrast-enhancing, hist-GBM: histologically confirmed glioblastoma, mol-GBM: molecular glioblastoma, OS: overall survival


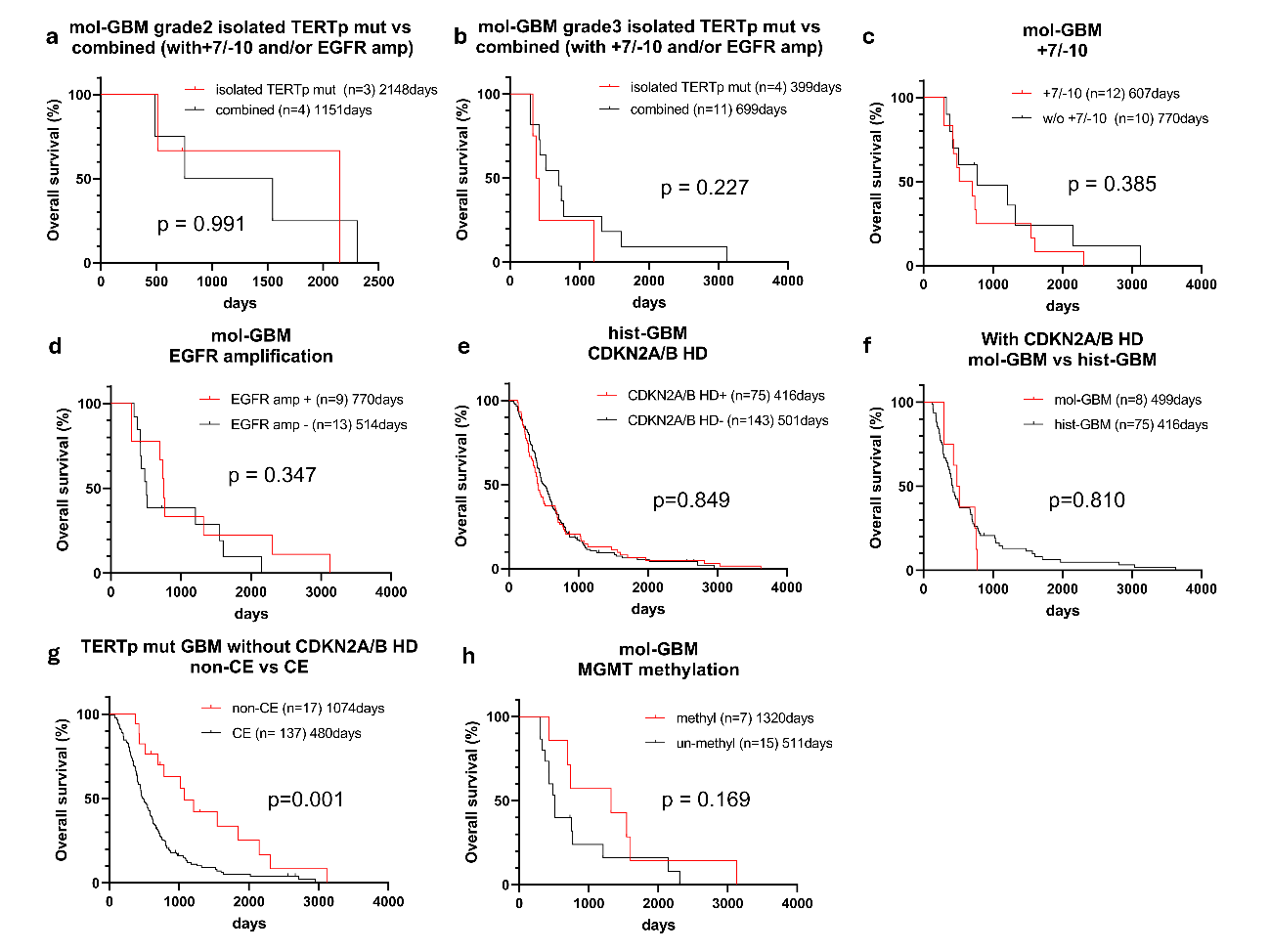


**Supplementary Fig. S2** **a, b** Kaplan–Meier survival curves comparing OS between patients with isolated *TERT*p mutation and those with combined alterations (*TERT*p mutation plus *EGFR* amplification and/or +7/−10), stratified by histological grade (a, grade 2; b, grade 3). **c** Kaplan–Meier survival curves of mol-GBM cases according to chromosome +7/−10 status. **d** Kaplan–Meier survival curves of mol-GBM according to *EGFR* amplification status. **e** Kaplan–Meier survival curves of hist-GBM according to *CDKN2A/B* HD status. **f** Kaplan–Meier survival curves comparing OS between mol-GBM and hist-GBM among cases with *CDKN2A/B* HD. **g** Kaplan–Meier survival curves comparing OS between non-CE and CE tumors among *TERT*p–mutant GBMs without *CDKN2A/B* HD. **h** Kaplan–Meier survival curves of mol-GBM cases according to *MGMT* promoter methylation status. CE: contrast-enhancing, hist-GBM: histologically confirmed glioblastoma, HD: homozygous deletion, mol-GBM: molecular glioblastoma, OS: overall survival, *TERT*p: *TERT* promoter
